# APOBEC-mediated mutagenesis is a favorable predictor of prognosis and immunotherapy for bladder cancer patients: evidence from pan-cancer analysis and multiple databases

**DOI:** 10.7150/thno.73235

**Published:** 2022-05-16

**Authors:** Run Shi, Xin Wang, Yang Wu, Bin Xu, Tianyu Zhao, Christian Trapp, Xuanbin Wang, Kristian Unger, Cheng Zhou, Shun Lu, Alexander Buchner, Gerald Bastian Schulz, Fengjun Cao, Claus Belka, Chuan Su, Minglun Li, Yongqian Shu

**Affiliations:** 1 Department of Oncology, The First Affiliated Hospital of Nanjing Medical University, Nanjing, China.; 2 Department of Oncology, Jiangsu Cancer Hospital, Jiangsu Institute of Cancer Research, The Affiliated Cancer Hospital of Nanjing Medical University, Nanjing, China.; 3 Pancreas Center, The First Affiliated Hospital of Nanjing Medical University, Nanjing, China.; 4 Department of Urology, Shanghai Ninth People's Hospital, Shanghai Jiaotong University School of Medicine, Shanghai, China.; 5 Institute and Clinic for Occupational, Social and Environmental Medicine, LMU University Hospital Munich; Comprehensive Pneumology Center (CPC) Munich, German Center for Lung Research (DZL), Munich, Germany; Institute of Epidemiology, Helmholtz Zentrum München - German Research Center for Environmental Health, Neuherberg, Germany.; 6 Department of Radiation Oncology, University Hospital, LMU Munich, Germany.; 7 Laboratory of Chinese Herbal Pharmacology, Department of Pharmacology, Renmin Hospital; Hubei Key Laboratory of Wudang Local Chinese Medicine Research, Hubei University of Medicine, Shiyan, Hubei, China.; 8 Research Unit Radiation Cytogenetics, Helmholtz Center Munich, German Research Center for Environmental Health GmbH, Neuherberg, Germany.; 9 Department of Radiation Oncology, Nanfang Hospital, Southern Medical University, Guangzhou, China.; 10 Departmentof Radiotherapy, Sichuan Cancer Hospital, School of Medicine, University of Electronic Science and Technology of China, Chengdu, China.; 11 Department of Urology, University Hospital, LMU Munich, Germany.; 12 Department of Oncology, Renmin Hospital, Hubei University of Medicine, Shiyan, Hubei, China.; 13 German Cancer Consortium (DKTK), Munich, Germany.; 14 Department of Pathogen Biology and Immunology, Jiangsu Province Key Laboratory of Pathogen Biology, Center for Global Health, Nanjing Medical University, Nanjing, China.

**Keywords:** APOBEC mutagenesis, Pan-cancer analysis, Immunotherapy, Prognosis

## Abstract

**Background:** The APOBEC (apolipoprotein B mRNA editing enzyme, catalytic polypeptide-like) family-mediated mutagenesis is widespread in human cancers. However, our knowledge of the biological feature and clinical relevance of APOBECs and APOBEC mutagenesis in cancers remains limited.

**Methods:** In this study, with a series of bioinformatic and statistical approaches, we performed a comprehensive analysis of multiple levels of data, including whole-exome sequencing (WES) and targeted next-generation sequencing (NGS), transcriptome (bulk RNA-seq and single-cell RNA-seq), immune signatures and immune checkpoint blockade (ICB) potential, patient survival and drug sensitivity, to reveal the distribution characteristics and clinical significance of APOBECs and APOBEC mutagenesis in pan-cancer especially bladder cancer (BLCA).

**Results:** APOBEC mutagenesis dominates in the mutational patterns of BLCA. A higher enrichment score of APOBEC mutagenesis correlates with favorable prognosis, immune activation and potential ICB response in BLCA patients. APOBEC3A and 3B play a significant role in the malignant progression and cell differentiation within the tumor microenvironment. Furthermore, using machine learning approaches, a prognostic APOBEC mutagenesis-related model was established and validated in different BLCA cohorts.

**Conclusions:** Our study illustrates the characterization of APOBECs and APOBEC mutagenesis in multiple cancer types and highlights its potential value as a promising biomarker for prognosis and immunotherapy in BLCA.

## Introduction

Tumorigenesis is caused by somatic mutations, which are mainly attributable to intrinsic and extrinsic factors, including DNA replication dysfunction, mutagenic exposures, enzymatic modification of DNA, and defective DNA repair, etc. 1. A more comprehensive knowledge of somatic mutations and mutational processes will deepen our understanding of tumor evolution, immune escape, and treatment resistance. In recent years, deep sequencing techniques have been developed to identify somatic mutations in cancer samples, and different algorithms have offered the probability of deciphering mutational signatures 2, 3. Notably, among various mutational signatures, the APOBEC (apolipoprotein B mRNA editing enzyme, catalytic polypeptide-like) family contributes a major source of the DNA modification in cancer genome, showing a specific mutational pattern: APOBEC mutagenesis.

The APOBEC family in humans consists of 11 members, namely APOBEC1, AID, APOBEC2, APOBEC3A-H (without 3E), and APOBEC4. Except APOBEC2 and C4, the other members possess an intrinsic ability to convert cytosine to uracil (C to U) in both single-stranded DNA (ssDNA) and cellular mRNA 4. APOBEC family members play an important role in the innate antiviral immunity, such as restriction of HIV-1, HBV and HPV 5-7. However, just as each coin has two sides, these enzymes can also deaminate cytosines in the host genome and generate C to T transition and C to G transversion in the TCW motif (W = A or T), namely APOBEC mutagenesis 8.

In recent years, an increasing number of researches have been focusing on the roles of APOBEC family members and APOBEC mutagenesis in cancers. The APOBEC mutagenesis pattern is widespread in human cancers and correlates with cancer evolution and malignant progression 9-12. Among APOBEC family, APOBEC3B is the most widely investigated member in various cancers. For example, upregulation of APOBEC3B was reported to predict worse survival in ovarian cancer 13 and associate with aggressive phenotypes in breast cancer 14, but predict better outcomes of immune checkpoint blockade (ICB) in non-small cell lung cancer (NSCLC) 15. In short, our knowledge of the APOBEC family and APOBEC mutagenesis in cancers is increasing, but still limited.

In this study, we surveyed the expression profiles of APOBEC family and distribution features of APOBEC mutagenesis in 9,765 tumor samples across 28 TCGA solid cancers. Then, we focused on bladder cancer for further investigation due to the major contribution of APOBECs to the total mutations and mutational patterns in bladder cancer. To further reveal the relationships and clinical significance of APOBECs and APOBEC mutagenesis, we performed a comprehensive analysis of multiple levels of data, including whole-exome sequencing (WES) and targeted NGS (MSK-IMPACT), transcriptome (bulk RNA-seq and single-cell RNA-seq), immune signatures and ICB potential, patient survival and drug sensitivity, using a series of bioinformatic and statistical approaches. Overall, our study provided a better understanding of the biological feature and clinical significance of APOBECs and APOBEC mutagenesis in cancers, and implications to ICB therapy for advanced bladder cancer.

## Materials and Methods

### Genomic data and clinical information

Transcriptome profiling data and clinical annotations of 28 solid tumors (ACC, BLCA, BRCA, CESC, CHOL, COAD, ESCA, GBM, HNSC, KICH, KIRC, KIRP, LGG, LIHC, LUAD, LUSC, MESO, OV, PAAD, PRAD, READ, SARC, SKCM, STAD, TGCT, THCA, THYM, and UCEC) were obtained from The Cancer Genome Atlas (TCGA, https://portal.gdc.cancer.gov/) using R package “TCGAbiolinks”, and transcriptome profiling data of donated normal tissues were obtained from the Genotype-Tissue Expression (GTEx) project. FPKM was transformed to TPM for impartial comparison. Three BLCA microarray datasets named GSE13507 16, GSE32894 17 and GSE48075 18 with cancer-specific survival (CSS) information were included for validation, and all raw CEL files were downloaded from Gene Expression Omnibus (GEO, https://www.ncbi.nlm.nih.gov/geo/). Probe IDs were mapped to gene symbols according to the annotation file, and the maximal measurement was selected as the gene expression value if one gene has multiple probes. In addition, one RNA-seq dataset named E-MTAB-4321 19 which contains transcriptome profiling data and progression-free survival information of 460 non-muscle invasive bladder cancer (NMIBC) was included for independent validation. All the microarray and RNA-seq data included in this study were normalized and log2 transformed as previously reported 20-22.

Three somatic mutation profiles of BLCA samples based on the whole-exome sequencing (WES) platform were obtained from TCGA 23, BGI-Shenzhen 24, and DFCI/MSKCC 25, respectively. A genomic profile of targeted NGS assay (MSK-IMPACT, 341/410 panels) and clinical details of 140 ICB-treated advanced BLCA samples were obtained from Samstein's study 26.

Using R package “IMvigor210CoreBiologies”, the transcriptome data and clinical activity of PD-L1 blockade with atezolizumab of 298 metastatic BLCA patients (IMvigor210, a phase 2 trial) were obtained from Mariathasan's study 27. In addition, transcriptome data and therapeutic response of 47 melanoma patients who received immunotherapies, including PD-1 and CTLA-4 blockade were obtained from Roh's study 28.

### Deciphering driver genes and mutational signatures in BLCA

Somatic mutation profile of TCGA-BLCA, which was identified using MuTect2, was sorted in the mutation annotation format (MAF) file. Among all mutation variants, Frame_Shift_Del, Frame_Shift_Ins, In_Frame_Del, In_Frame_Ins, Missense, Nonsense, Nonstop, Splice_Site, and Translation_Start_Site were considered non-synonymous, and the other mutations were considered synonymous. Oncoplot was visualized based on the MAF file using R package “maftools” 29. The OncodriveCLUST algorithm 30 was used to explore driver genes with a significant bias toward mutation clustering within the protein sequence, and we defined that the significance of identified driver genes should meet false discovery rete (FDR) q < 0.05.

R package “Sigminer” proposed by Wang 31 was used to extract mutational signatures from the WES data. Bayesian variant of nonnegative matrix factorization (NMF) algorithm was used to decipher mutational signatures in cancer somatic mutations stratified by 96 base substitutions in trinucleotide sequence contexts, and the optimal factorization of k value is selected when the magnitude of the cophenetic correlation coefficient begins to drop significantly. The gradient boosting machine learning technique yields an individual score for each mutational signature, combining likelihood with cosine similarity and exposure of signatures using the NNLS algorithm. Mutational signatures were annotated by computing cosine similarity against validated single base substitution (SBS) mutational catalogues retrieved from the COSMIC database (v3.2) 32.

### Calculation of the APOBEC mutagenesis enrichment score (AMES)

APOBECs deaminate cytidines predominantly in a TCW motif (W = A or T). The APOBEC mutagenesis signature is composed of two mutation patterns in this motif: TCW to TTW and TCW to TGW. The quantitative score of APOBEC mutagenesis enrichment (AMES) which reflects the strength of such mutagenesis in the TCW motif was defined by Roberts et al. 9:



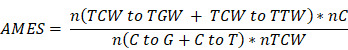



where n(TCW to TGW + TCW to TTW) is the number of mutated C (and G) falling in a TCW (or WGA) motif, n(C to G + C to T) is the total number of mutated C (or G), nC and nTCW represent the numbers of background cytosines and TCWs that occur within 20bp of mutated bases, respectively. In this study, tumor samples were categorized into three levels with two cut-off values of 1 and 2:



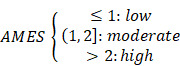



### Evaluating variable importance using the random forest (RF) algorithm

The random forest (RF) algorithm was applied to evaluate the contributions of all 11 APOBEC family members to TCW motif mutation and AMES in pan-cancer samples, as well as the importance of different cancer hallmarks in malignant epithelial cell subclusters. A total of 1,000 decision trees were created in the RF algorithm to ensure the model stability.

### Single-cell RNA-seq (scRNA-seq) analysis

Two scRNA-seq datasets (GSE130001 33 and GSE145281 34) were included in this study to reveal the expression characteristics of APOBECs in BLCA and its tumor microenvironment (TME). GSE130001 includes scRNA-seq data of sorted non-immune cells (CD45-negative) from two BLCA specimens, and GSE145281 includes scRNA-seq data of baseline pretreatment peripheral blood mononuclear cell (PBMC) samples from five BLCA patients who are responders to atezolizumab (anti-PD-L1 mAb). The scRNA-seq expression matrix was processed with R package “Seurat”. At first, the “NormalizeData” function was used to normalize the gene expression data, and “FindVariableFeatures” was used to identify 2,000 highly variable genes (HVGs). After performing “RunPCA” for dimension reduction and “RunHarmony” for batch effect correction, “FindNeighbors” was used to determine the k-nearest neighbors of each cell, and “FindClusters” was used to determine optimal clusters. UMAP reduction was used for cluster visualization, and “SingleR” package was used for cluster annotation. In addition, “FeaturePlot” and “VlnPlot” were used to visualize gene expression.

Using the Monocle2 algorithm with default parameters, the pseudotime trajectory analysis was performed to arrange cells into a developmental trajectory which was segmented with different branches to imitate cell evolution or differentiation.

To identify malignant epithelial cells, R package “infercnv” was used to infer CNVs with scRNA-seq data, and the following parameters were used: default denoise and Hidden Markov Model (HMM) settings, and a “cutoff” of 0.1 for 10× genomics. Stromal cells including endothelial cells, fibroblasts, and myofibroblasts were considered as putative non-malignant cells, and their CNV pattern was used as a reference. CNVs of sex and mitochondrial chromosomes were excluded from consideration.

### Gene set enrichment analysis (GSEA) and single-sample GSEA (ssGSEA)

Based on the transcriptome profiling data and gene sets retrieved from Molecular Signatures Database (MSigDB) 35, GSEA 36 was performed to analyze the potential signaling pathways between two groups. Furthermore, the fast GSEA algorithm was implemented to generate an integrative enrichment plot which exhibits the 10 most significant biological processes of Gene Ontology (GOBP) using R package “fgsea” 37.

A T cell-inflamed GEP composed of 18 inflammatory genes were used to evaluate the potential response of ICB treatment in different groups 38, 39. Furthermore, the T cell-inflamed score was calculated with the transcriptome profiling data using the ssGSEA algorithm (R package “GSVA”) 40.

### Analyses of immunogenomic features

Tumor mutation burden (TMB) was calculated with non-synonymous somatic mutations using 38 Mb as the estimation of whole exome size. Neoantigens of TCGA BLCA samples were obtained from The Cancer Immunome Atlas (TCIA) 41, and the tumor neoantigen burden (TNB) was determined as the amount of all putative neoantigens. The intra-tumor heterogeneity (ITH) score was inferred using the “inferHeterogeneity” function in the R package “maftools”. The expression profiles of four representative immune checkpoints (PD-1, PD-L1, CTLA-4, and TIGIT) were compared in different AMES groups. Cytolytic activity (CYT) score was defined as the geometric mean of PRF1 and GZMA 42.

### Analysis of the DNA damage response (DDR) status

Samples with any non-silent mutation in any DDR pathway, including base excision repair (BER), nucleotide excision repair (NER), mismatch repair (MMR), homologous recombination repair (HRR), non-homologous DNA end joining (NHEJ), Fanconi anemia (FA), and translesion synthesis (TLS) were considered DDR-mutated (DDR-Mut), while the others were considered DDR-wild type (DDR-WT). Furthermore, if any gene involved in a specific DDR pathway is non-silently mutated, this DDR pathway will be regarded as a mutated one.

### Estimation of immune cell infiltration

With transcriptome profiling data, ESTIMATE algorithm 43 was used to estimate the immune infiltration, and CIBERSORT algorithm 44 was used to quantify the abundance of 22 immune cell types. Based on the expression profile of APOBEC family members and estimated immune infiltration abundance, a landscape diagram depicting the correlations between APOBECs and seven major-lineage immune cell types (including B cell, T cell, NK cell, mast cell, monocyte, M1 and M2 macrophages) was generated using R package “ggcor”.

### Identification of differentially expressed genes (DEGs) and functional network analysis

DEGs were identified with a threshold of a false discovery rate (FDR) q < 0.01 based on reads count matrix and R package “DESeq2”. Functional enrichment analysis of DEGs was performed using R package “clusterProfiler”. Protein-protein interaction (PPI) network which depicts the interactions among representative DEGs was generated using R package “STRINGdb” and “ggraph” as previously reported 21.

### Establishment and validation of a prognostic APOBEC mutagenesis-related risk score (AMrs)

Using R package “coxph”, hazard ratio and p value were calculated for each DEG, and candidate genes with p < 0.01 were further submitted to the least absolute shrinkage and selection operator (LASSO) Cox regression analysis as previously reported 20, 21. LASSO regularization adds a penalty parameter (λ) to Cox regression model, and this action can lead to zero coefficients, i.e. some candidate genes will be completely neglected. In our analysis, 21 genes retained their Cox coefficients after LASSO regularization, and a prognostic APOBEC mutagenesis-related risk score (AMrs) was established:



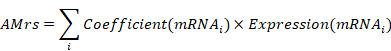



Further, two independent BLCA cohorts (GSE13507 and GSE32894) with cancer-specific follow-up were used to validate the prognostic value of AMrs.

### Drug sensitivity estimation

Drug sensitivity data of cancer cell lines (CCLs) were obtained from three drug response databases, namely GDSC 45, CTRP 46, and PRISM 47. Both CTRP and PRISM contain the AUC value as a measure of drug sensitivity, and GDSC contains the IC50 value. Lower IC50 (or AUC) value indicates a higher sensitivity to compound treatment. Transcriptome profiling data of CCLs were obtained from the CCLE database 48. The IC50 value for each compound from GDSC was estimated using R package “oncoPredict” 49. For CTRP and PRISM, compounds with over 20% missing data were excluded before KNN imputation, and the “calcPhenotype” function of the R package “pRRophetic” was used to predict the AUC value for each compound following Yang's methods 50. Spearman correlation coefficient of AMrs and IC50 (or AUC) represents the potential response of a BLCA sample to a specific compound.

### Statistical analyses

Heatmaps were generated using R package “pheatmap”. Differentially mutated genes and the co-occurrence or exclusion of somatic mutations were identified by Fisher's exact test. A ternary diagram was plotted to show the mutation frequency among three AMES groups. Pearson or Spearman correlation analysis was performed to evaluate the correlation between two variables. The Kaplan-Meier method was used to plot survival curves, and the log-rank test was performed to evaluate survival differences. Multivariate Cox regression analysis was performed to evaluate the risk significance of each variable for prognosis. The Subclass Mapping (SubMap) method was applied to evaluate the expression similarity between independent datasets, and the significance was evaluated with the Bonferroni correction. Student's t-test or one-way analysis of variance was used to analyze differences between groups with variables subject to normal distribution. Two-sided p value less than 0.05 was considered statistically significant. All analyses were performed in the GenePattern and R 4.1.0 software.

## Results

### Landscape of APOBEC family expression and APOBEC mutagenesis in pan-cancer

**Figure 1:**
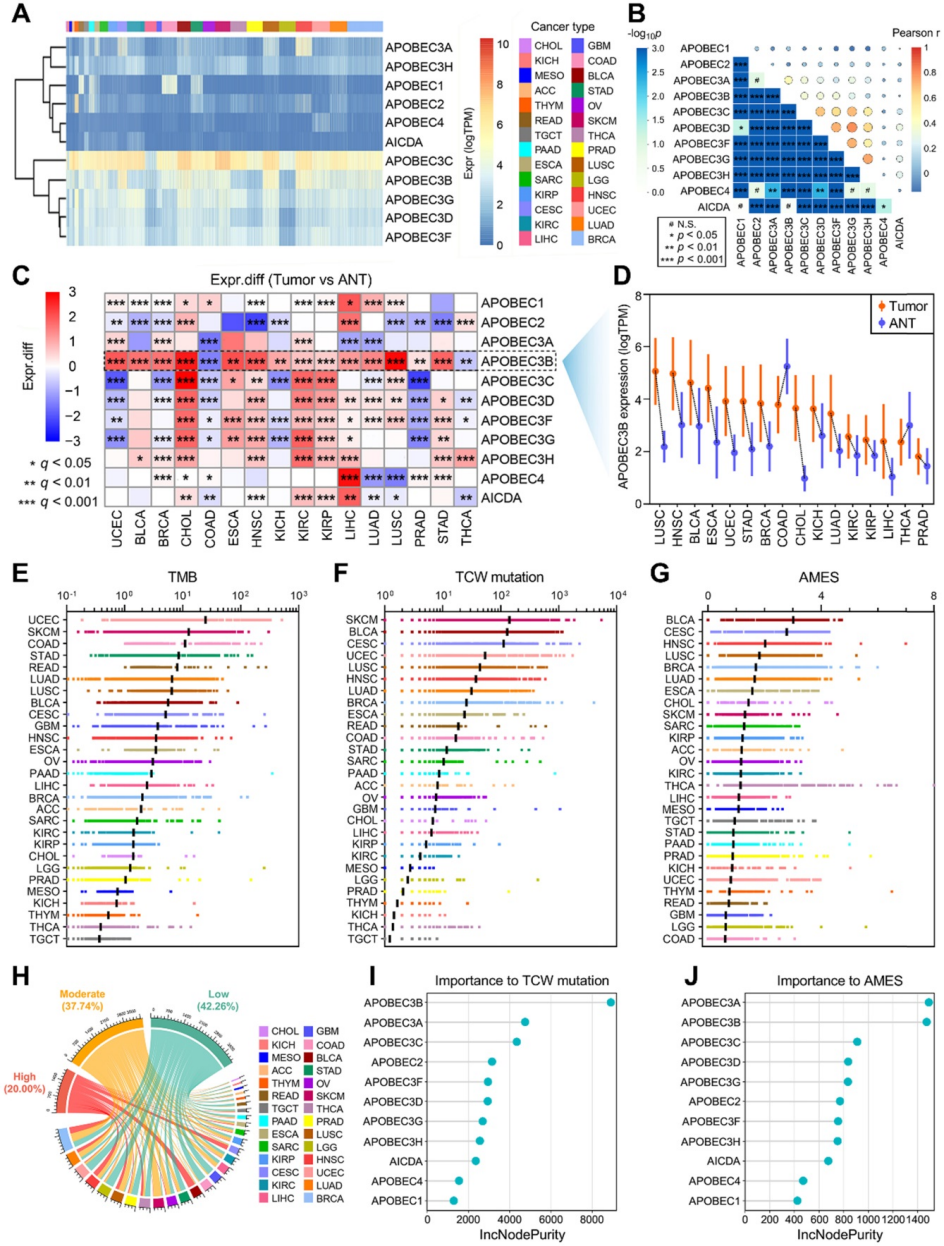
** Landscape of APOBEC family expression and APOBEC mutagenesis in pan-cancer. (A)** A comprehensive heatmap illustrates the expression pattern of APOBECs across 28 solid cancer types from TCGA. **(B)** The Pearson correlation analysis was performed among all 11 APOBEC family members in pan-cancer. **(C)** All 11 APOBEC family members were compared in primary tumors versus adjacent normal tissues (ANT). **(D)** Among all the APOBECs, only APOBEC3B exhibited dysregulation across all 16 cancer types. Compared to ANTs, APOBEC3B was significantly downregulated in COAD and THCA, while upregulated in the other 14 cancer types. **(E-G)** Distribution landscape of the TMB, TCW mutation count and APOBEC mutagenesis enrichment score (AMES) in all 28 cancer types. **(G)** BLCA shows the highest AMES among all 28 cancer types. **(H)** With two cut-off values of 1 and 2, the total of 9,550 pan-cancer samples were divided into three levels: AMES-low (AMES-L, 42.26%), -moderate (AMES-M, 37.74%), and -high (AMES-H, 20.00%). **(I)** The random forest algorithm showed that in pan-cancer, APOBEC3B is the most important contributor to TCW mutation, **(J)** and both APOBEC3A and APOBEC3B make the greatest contributions to AMES.

To investigate the genomic features of the 11 genes of APOBEC family in pan-cancer, a comprehensive heatmap was generated to illustrate their expression patterns in a total of 9,765 samples (including 9,398 primary tumors and 367 metastatic tumors) across 28 solid cancer types from TCGA (Figure 1A). Among all the APOBECs, APOBEC3C showed the highest expression in pan-cancer. We also observed that some APOBECs exhibit a specifically high expression in some certain cancer types, e.g. APOBEC2 in THYM. The Pearson correlation analysis was performed among all 11 APOBEC family members, and most pairs exhibited significantly positive correlations, especially among APOBEC3s (Figure 1B). We further obtained 16 cancer types which have at least five specimens of adjacent normal tissue (ANT) and assessed the expression profiles of 11 APOBEC family members by comparing them in primary tumors versus ANTs. As shown in Figure 1C, APOBECs were widely dysregulated across the 16 cancer types compared to their corresponding ANTs. Most notably, among all the APOBECs, only APOBEC3B exhibited dysregulation across all 16 cancer types. Compared to ANTs, APOBEC3B was significantly downregulated in COAD and THCA, while upregulated in the other 14 cancer types. Details of the expression profile of APOBEC3B in the 16 cancer types and ANTs were shown in Figure 1D.

Next, we calculated the tumor mutation burden (TMB), TCW mutation (including TCW to TTW and TCW to TGW, where W means A or T) count and APOBEC mutagenesis enrichment score (AMES) for each tumor sample across all 28 cancer types. UCEC, SKCM, and COAD occupy the top three positions in the TMB ranking (Figure 1E), and SKCM, BLCA, and CESC as the top three in the TCW mutation count ranking (Figure 1F). After mutation background adjustment, AMES was calculated to quantify the APOBEC mutagenesis enrichment, and we observed that BLCA shows the highest AMES among all 28 cancer types (Figure 1G). With two cut-off values of 1 and 2, the total of 9,550 pan-cancer samples were divided into three levels: AMES-low (AMES-L, 42.26%), -moderate (AMES-M, 37.74%), and -high (AMES-H, 20.00%) (Figure 1H). Random forest algorithm showed that in pan-cancer, APOBEC3B acts as the most important contributor to TCW mutation (Figure 1I), and both APOBEC3A and APOBEC3B make the greatest contributions to AMES (Figure 1J). AMES of pan-cancer samples were summarized in Supplementary Table 1.

### Distinct mutational characteristics were observed in different AMES groups

**Figure 2:**
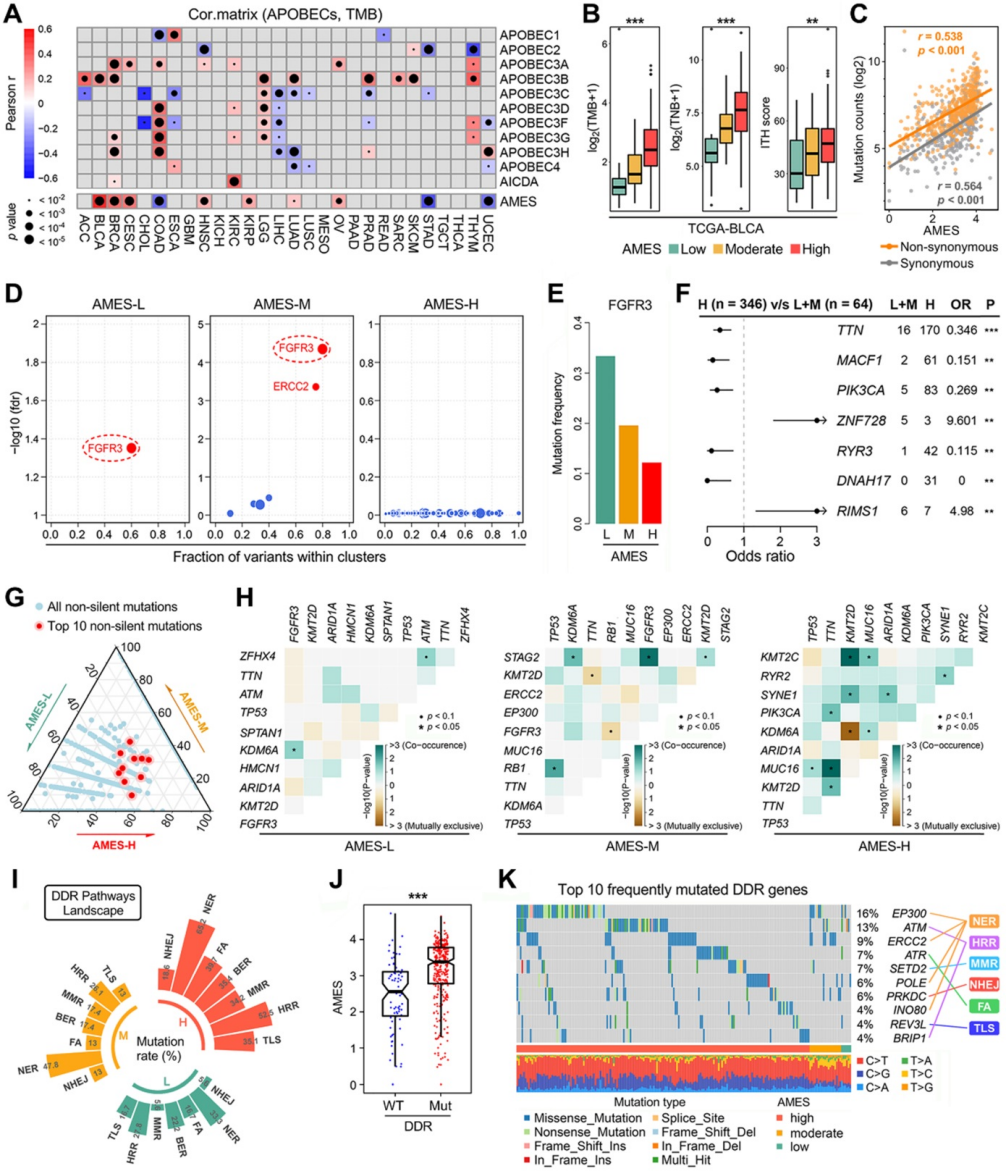
** Higher AMES correlates with higher TMB, intra-tumor heterogeneity and DDR mutation frequency in BLCA. (A)** The correlations among TMB, AMES, and APOBECs were analyzed in pan-cancer. Among all cases, AMES exhibited the highest and most significant correlation with TMB in BLCA (r = 0.514, p < 2.2e-16). **(B)** TMB, TNB, and ITH score were stepwisely and significantly elevated as AMES increased. **(C)** AMES showed highly positive correlations with both non-synonymous (r = 0.538, p < 0.001) and synonymous (r = 0.564, p < 0.001) mutations in BLCA samples. **(D)** FGFR3 is a mutual driver gene in both AMES-L and AMES-M groups, while no driver gene was detected in the AMES-H group. **(E)** FGFR3 mutation frequency descends as AMES level increases. **(F)** Significantly mutated genes were identified in the AMES-H group compared to the other two groups, and TTN, MACF1 and PIK3CA are the three most frequently mutated genes in AMES-H samples. ** p < 0.01; *** p < 0.001. **(G)** A ternary diagram was plotted to depict the distribution of all non-silent mutations in the three AMES groups, and the 10 most frequent non-silent mutations (TP53, TTN, KMT2D, MUC16, KDM6A, ARID1A, PIK3CA, SYNE1, RB1, and KMT2C) were highlighted in red dots. **(H)** More co-occurrence and mutually exclusive mutations could be observed as AMES level increased. **(I)** Mutation rates of seven DDR pathways (BER, NER, MMR, HRR, NHEJ, FA, and TLS) were summarized in different AMES groups, and a higher frequency of DDR pathway mutation was observed in the AMES-H group. **(J)** AMES is significantly elevated in DDR-Mut samples compared to DDR-WT. **(K)** An oncoplot shows the distribution of the 10 most frequently mutated DDR genes among different AMES groups.

Considering TMB serves as an important index for mutational analysis and cancer immunotherapy, we assessed the correlations among TMB, AMES, and APOBECs in pan-cancer. In the comprehensive heatmap (Figure 2A), the color degree indicates the correlation, and the point size indicates the significance. Among all cases, AMES exhibited the highest and most significant correlation with TMB in BLCA (r = 0.514, p < 2.2e-16; Figure 2A). Next, we compared TMB, TNB, and ITH among different AMES levels. As shown in Figure 2B, TMB, TNB, and ITH score were stepwisely and significantly elevated as AMES increased, which suggested that AMES may correlate with higher immunogenicity and heterogeneity in BLCA. Furthermore, AMES showed highly positive correlations with both non-synonymous (r = 0.538, p < 0.001) and synonymous (r = 0.564, p < 0.001) mutations in BLCA samples (Figure 2C).

The OncodriveCLUST algorithm indicated that FGFR3 acts as a mutual driver gene in both AMES-L and AMES-M groups, while no driver gene was detected in the AMES-H group (Figure 2D). The barplot further demonstrated that FGFR3 mutation frequency descends as AMES level increases (Figure 2E). Significantly mutated genes were identified in the AMES-H group compared to the other two groups. As shown in a forest plot (Figure 2F), TTN, MACF1 and PIK3CA are the three most frequently mutated genes in AMES-H samples. A ternary diagram was plotted to depict the distribution of all non-silent mutations in the three AMES groups, and the 10 most frequent non-silent mutations (TP53, TTN, KMT2D, MUC16, KDM6A, ARID1A, PIK3CA, SYNE1, RB1, and KMT2C; Figure S1) were highlighted in red dots (Figure 2G). We observed that the top 10 mutated genes have a tendency towards occurrence in the AMES-H group (Figure 2G). In addition, more co-occurrence and mutually exclusive mutations could be observed as AMES level increased, which indicated that AMES correlates with higher somatic mutation activity in BLCA (Figure 2H).

DNA damage response (DDR) genes are commonly mutated in BLCA, and alterations to DDR genes were associated with favorable ICB outcomes in BLCA patients 51. Mutation rates of seven DDR pathways (BER, NER, MMR, HRR, NHEJ, FA, and TLS) were summarized in different AMES groups (Figure 2I). In BLCA, NER is the most frequently mutated DDR pathway, and HRR comes second. A higher frequency of DDR pathway mutation was observed in the AMES-H group (Figure 2I), and obviously, AMES is significantly elevated in DDR-Mut samples compared to DDR-WT (Figure 2J). An oncoplot was built to display the 10 most frequently mutated DDR genes among different AMES groups (Figure 2K). In detail, EP300, ERCC2, POLE, and INO80 are involved in the NER pathway, ATM and BRIP1 in HRR, ATR in FA, SETD2 in MMR, PRKDC in NHEJ, and REV3L in TLS.

### APOBEC mutagenesis dominates in the mutational patterns of BLCA

**Figure 3:**
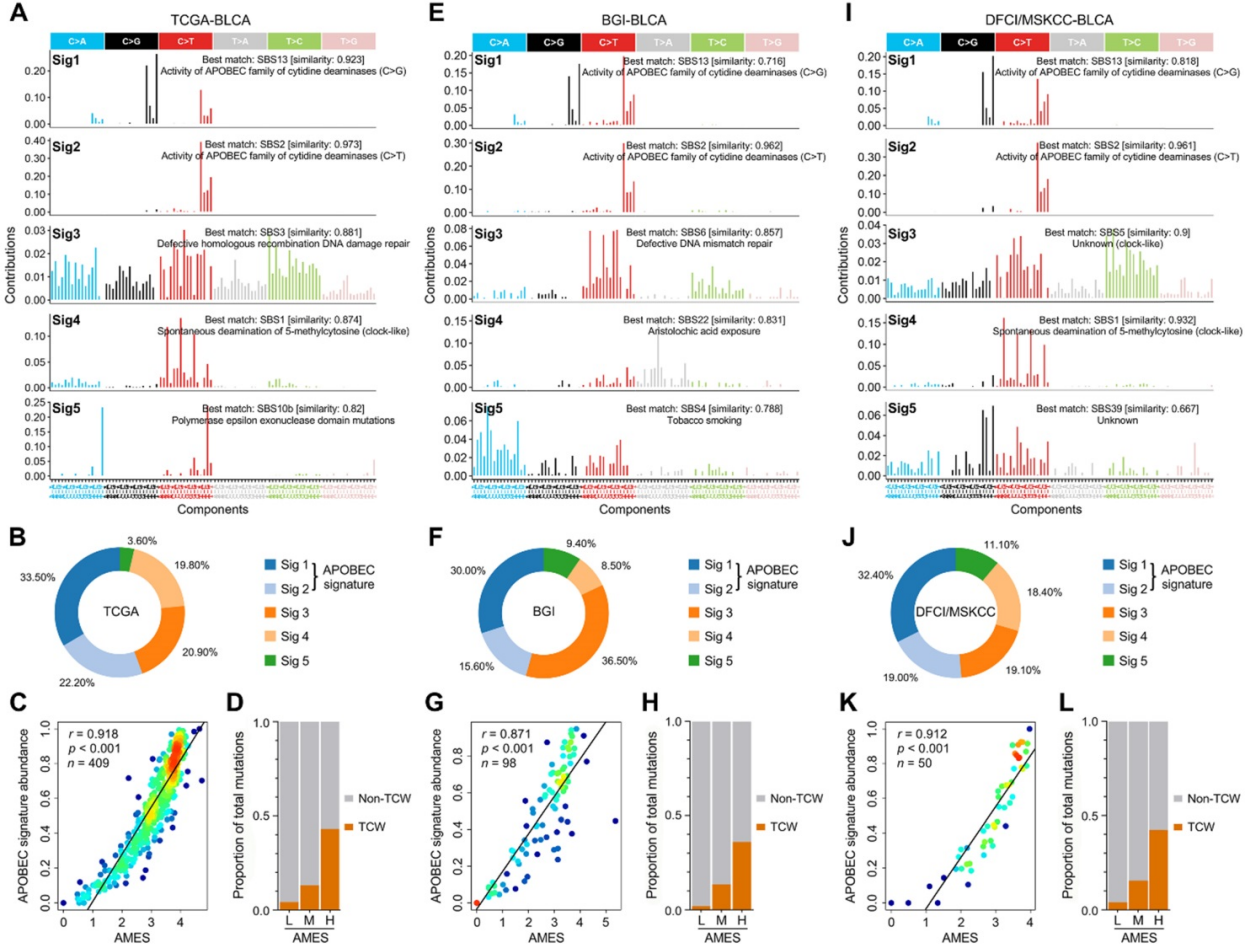
** APOBEC mutagenesis dominates in the mutational patterns of BLCA.** To decipher mutational signatures in BLCA, three WES cohorts (TCGA-BLCA, BGI-BLCA, and DFCI/MSKCC-BLCA) were included in our study. **(A)** With the optimal factorization k value (k = 5) in the NMF algorithm, five mutational signatures were identified in the TCGA-BLCA cohort. Signature 1 was annotated as “activity of APOBEC family of cytidine deaminases (C>G)”, and signature 2 “activity of APOBEC family of cytidine deaminases (C>T)”. **(B)** The relative abundance of each mutational signature in the TCGA cohort was shown in a pie chart, and APOBEC signatures (Sig1 + 2) occupy a dominant position. **(C)** APOBEC signature abundance shows a highly positive correlation with AMES, **(D)** and TCW mutations were dramatically elevated as AMES level increased. Similar results were observed in both **(E-H)** BGI-BLCA and **(I-L)** DFCI/MSKCC-BLCA cohorts, which further confirmed that APOBEC mutagenesis dominates in BLCA mutational patterns and retains a highly positive correlation with AMES.

To decipher mutational signatures in BLCA, we included three WES cohorts in our study. With the optimal factorization k value (k = 5; Figure S2) in the NMF algorithm, five mutational signatures were identified in the TCGA-BLCA cohort. Signature 1 was annotated as “activity of APOBEC family of cytidine deaminases (C>G)”, and signature 2 “activity of APOBEC family of cytidine deaminases (C>T)” (Figure 3A). The relative abundance of each mutational signature in the TCGA cohort was shown in a pie chart (Figure 3B), and we can easily find that APOBEC signatures (Sig1 + 2) retain a dominant position. In addition, in the TCGA-BLCA cohort, APOBEC signature abundance shows a highly positive correlation with AMES (Figure 3C), and TCW mutations were dramatically elevated as AMES level increased (Figure 3D). Interestingly, similar results were observed in both BGI-BLCA (Figure 3E-H) and DFCI/MSKCC-BLCA (Figure 3I-L) cohorts. In brief, APOBEC mutagenesis dominates in BLCA mutational patterns, and retains a highly positive correlation with AMES.

### APOBEC3B correlates with malignant evolution of epithelial cells in BLCA

**Figure 4:**
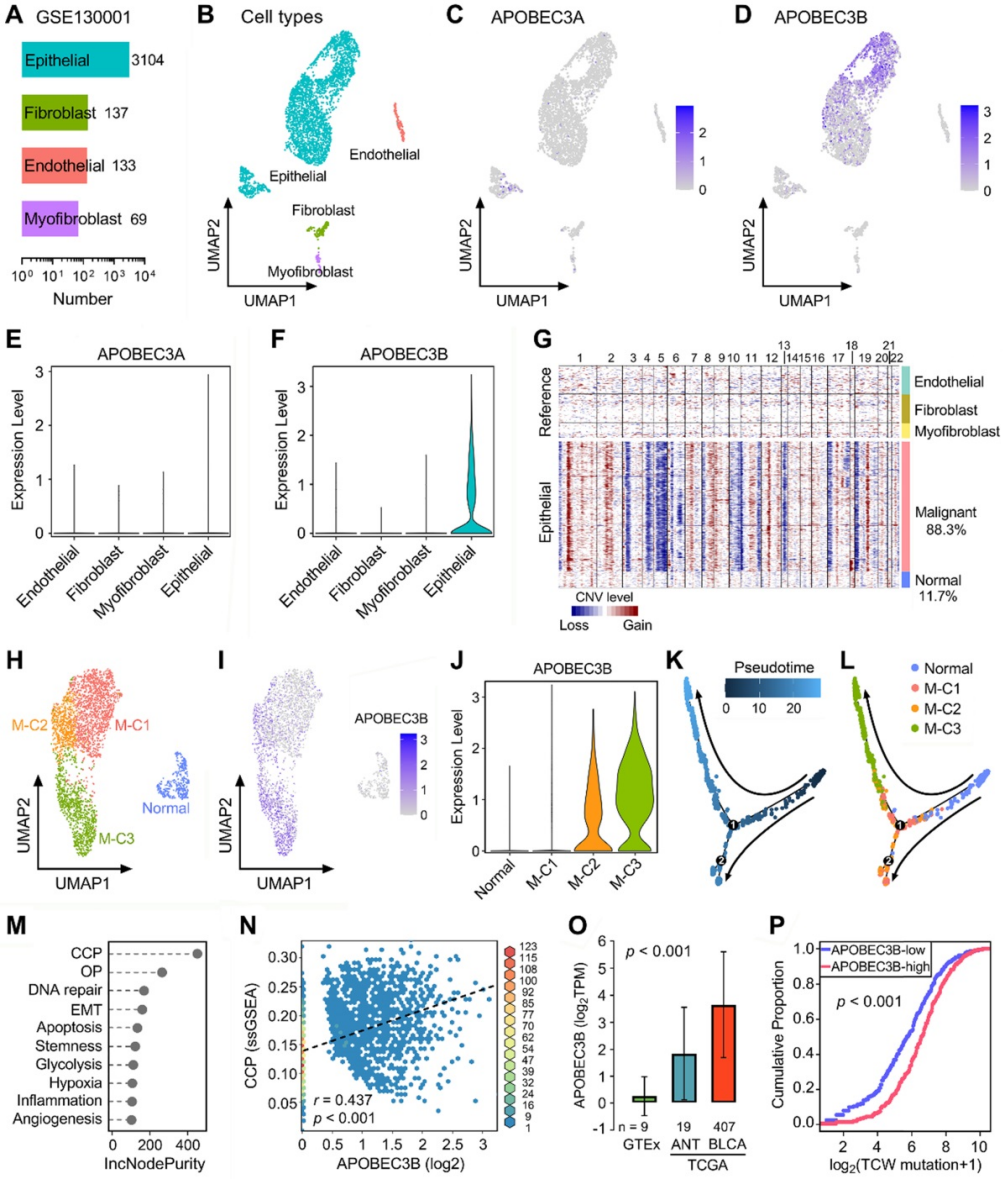
** APOBEC3B is specifically expressed in malignant epithelial cells and correlates with cell cycle progression in BLCA. (A)** GSE130001 contains scRNA-seq data of sorted non-immune cells from two BLCA specimens, and cell numbers of four cell types (epithelial, fibroblast, endothelial, and myofibroblast) were summarized. **(B)** UMAP dimensionality reduction was used to show the distribution and dissimilarity of the four cell types. **(C)** APOBEC3A is hardly expressed in any cell type, **(D)** while APOBEC3B is specifically expressed in epithelial cells. **(E & F)** Violin plots visualized the normalized expression levels of APOBEC3A and APOBEC3B in the four cell types. **(G)** Malignant cells were distinguished from the total epithelial cells by inferring large-scale CNVs with stromal cells as references, and 88.3% epithelial cells were identified as malignant due to their high chromosome instability. **(H)** Using UMAP dimensionality reduction, malignant epithelial cells were further divided into three subclusters (M-C1, M-C2, M-C3), **(I)** and the expression of APOBEC3B was visualized with different color degrees. **(J)** Violin plot showed that APOBEC3B is hardly expressed in normal epithelial cells and M-C1, but highly expressed in M-C2 and M-C3. **(K)** A pseudotime trajectory was plotted to describe the evolution of epithelial cells, and the arrows indicated the putative developmental directions. **(L)** The progression trajectory originated from normal epithelial cells and developed into two main branches where M-C3 cells located at the top-left corner and M-C1 & C2 located at the lower-left corner. **(M)** The ssGSEA and random forest algorithms jointly demonstrated that cell cycle progression (CCP) acts as the most important hallmark of cancer among the three subclusters. OP: oxidative phosphorylation. **(N)** In all malignant epithelial cells, APOBEC3B exhibited a significantly positive correlation with CCP score (r = 0.437, p < 0.001). **(O)** APOBEC3B was stepwisely and significantly elevated as specimens from normal bladder tissues to ANT, and to BLCA (p < 0.001). **(P)** Cumulative proportion curves showed that the higher-APOBEC3B group (red curve) was continuously distributed at the right side of the lower-APOBEC3B group (blue curve), indicating APOBEC3B contributes substantially to TCW mutations in BLCA.

Next, we included two scRNA-seq datasets (GSE130001 and GSE145281) to further reveal the expression profiles of APOBECs in BLCA and its TME. GSE130001 contains scRNA-seq data of sorted non-immune cells from two BLCA specimens, and cell numbers of four cell types (epithelial, fibroblast, endothelial, and myofibroblast) were summarized in Figure 4A. UMAP dimensionality reduction was used to show the distribution and dissimilarity of the four cell types (Figure 4B). Subsequently, we assessed the expression profiles of all 11 APOBEC family members in the four cell types (Figure S3). As the two most important APOBECs in BLCA, APOBEC3A is hardly expressed in any cell type (Figure 4C), while APOBEC3B is specifically expressed in epithelial cells (Figure 4D). Violin plots visualized the normalized expression levels of APOBEC3A and APOBEC3B in the four cell types (Figure 4E-F). Malignant cells were distinguished from the total epithelial cells by inferring large-scale CNVs with stromal cells as references, and 88.3% epithelial cells were identified as malignant due to their high chromosome instability (Figure 4G). Using UMAP dimensionality reduction, malignant epithelial cells were further divided into three subclusters (M-C1, M-C2, M-C3; Figure 4H), and the expression of APOBEC3B was visualized with different color degrees (Figure 4I). Violin plot showed that APOBEC3B is hardly expressed in normal epithelial cells and M-C1, but highly expressed in M-C2 and M-C3 (Figure 4J). Next, we performed pseudotime trajectory analysis to describe the evolution of epithelial cells, and the arrows indicated the putative developmental directions (Figure 4K). The progression trajectory originated from normal epithelial cells and developed into two main branches where M-C3 cells located at the top-left corner and M-C1 & C2 located at the lower-left corner (Figure 4L). To elucidate the underlying biological diversities in different malignant subclusters, we combined ssGSEA and random forest algorithms to determine which hallmark of cancer plays a distinctive role among the three malignant subclusters. Among various cancer hallmarks, cell cycle progression (CCP) acts as the most important one (Figure 4M; “OP” is short for “oxidative phosphorylation”). In all malignant epithelial cells, APOBEC3B exhibited a significantly positive correlation with CCP score (r = 0.437, p < 0.001; Figure 4N). Moreover, APOBEC3B was stepwisely and significantly elevated as specimens from normal bladder tissues to ANT, and to BLCA samples (p < 0.001; Figure 4O). Cumulative proportion curves showed that the higher-APOBEC3B group (red curve) was continuously distributed at the right side of the lower-APOBEC3B group (blue curve), indicating APOBEC3B contributes substantially to TCW mutations in BLCA (Figure 4P).

### APOBEC3A correlates with the differentiation fate of monocytes in BLCA

**Figure 5:**
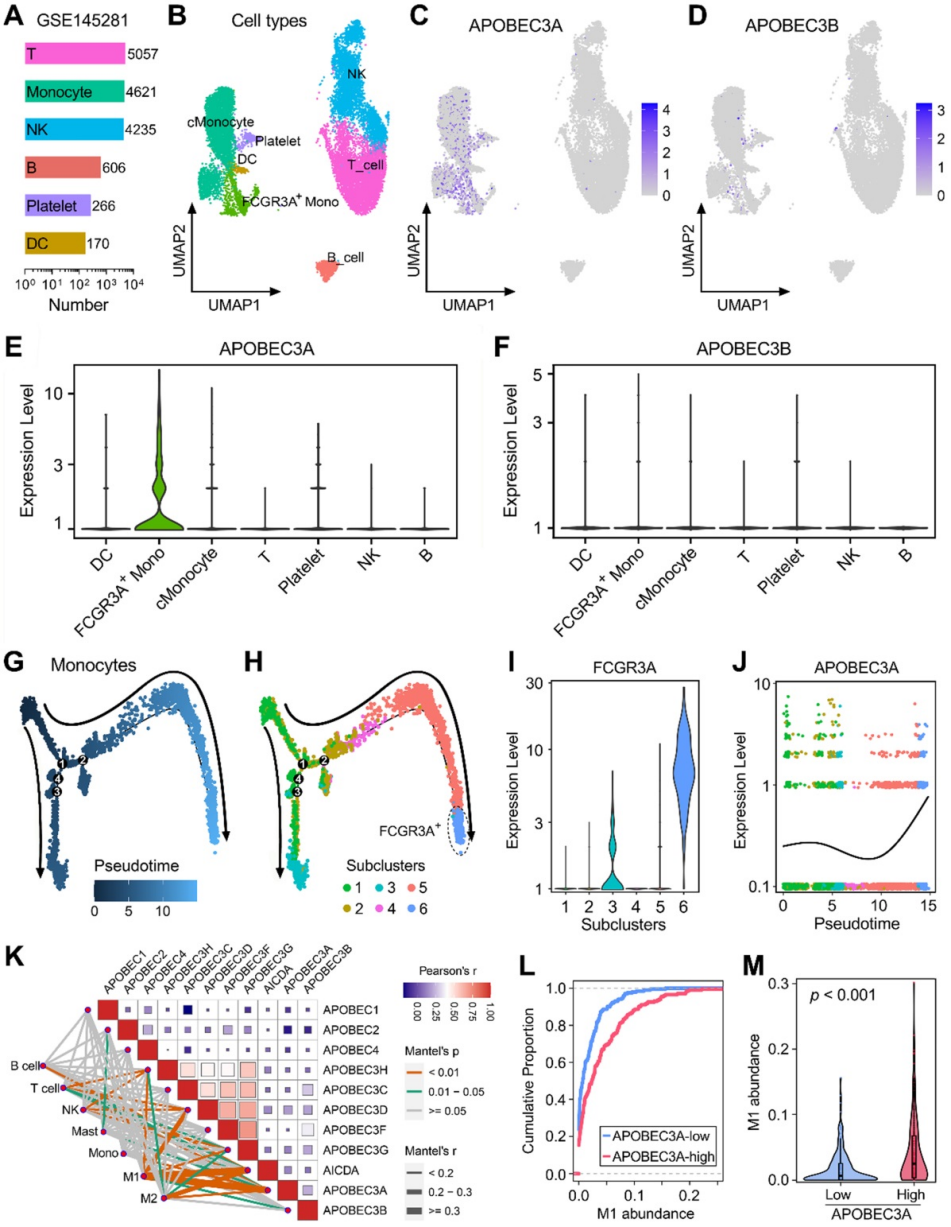
** APOBEC3A correlates with the differentiation fate of monocytes in BLCA. (A)** GSE145281 contains scRNA-seq data of baseline pretreatment PBMC samples from BLCA patients, and cell numbers of six cell types (T, monocyte, NK, B, Platelet, and DC) were summarized. **(B)** UMAP dimensionality reduction was performed to show the distribution and dissimilarity of these cell types. **(C)** APOBEC3A is generally expressed in monocytes, **(D)** while APOBEC3B is hardly expressed in PBMC. **(E & F)** Violin plots showed the normalized expression levels of APOBEC3A and APOBEC3B in all identified cell types, and APOBEC3A is specifically expressed in FCGR3A+ monocytes. **(G)** All monocytes were included to perform the pseudotime trajectory analysis, and the arrows indicated the developmental directions. **(H)** Monocytes were clustered into six subclusters after UMAP dimensionality reduction, and two main branches ended with subcluster 3 (cyan) and 6 (lightblue). **(I)** As a canonical gene marker for identification of monocyte subsets, FCGR3A was compared among all six subclusters. Subcluster 3 expressed intermediate FCGR3A, subcluster 6 expressed the highest FCGR3A, and little FCGR3A was detected in the remaining four subclusters. **(J)** In the dynamic expression profile of APOBEC3A in monocyte pseudotime trajectory, APOBEC3A became highly expressed at the final stage which is labelled with FCGR3A positive. **(K)** A correlation heatmap illustrates the relationships among APOBECs and different immune cell infiltration (absolute abundance, CIBERSORT algorithm) in bulk BLCA samples. Among all cases, the pair of APOBEC3A-M1 exhibited the highest correlation (r = 0.334, p = 5.09e-12). **(L)** The cumulative proportion curves and **(M)** violin plot jointly demonstrated that a significantly higher abundance of M1 infiltration was observed in BLCA samples with higher APOBEC3A expression.

GSE145281 contains scRNA-seq data of baseline pretreatment PBMC samples from BLCA patients, and cell numbers of six cell types (T, monocyte, NK, B, Platelet, and DC) were summarized in Figure 5A. UMAP dimensionality reduction was performed to show the distribution and dissimilarity of these cell types (Figure 5B). Subsequently, the expressions of APOBEC family members were detected in these cell types (Figure S4). Expressions of APOBEC3A and APOBEC3B were visualized in UMAP plots, and we observed that APOBEC3A is generally expressed in monocytes (Figure 5C), while APOBEC3B is hardly expressed in PBMC (Figure 5D). Violin plots showed the normalized expression levels of APOBEC3A and APOBEC3B in all identified cell types (Figure 5E-F), and we found that APOBEC3A is specifically expressed in FCGR3A+ monocytes (Figure 5E), rather than classical monocytes (cMonocytes). Subsequently, all monocytes were included to perform the pseudotime trajectory analysis, and the arrows indicated the developmental directions (Figure 5G). Monocytes were clustered into six subclusters after UMAP dimensionality reduction, and two main branches ended with subcluster 3 (cyan) and 6 (lightblue) (Figure 5H). As a canonical gene marker for identification of monocyte subsets, FCGR3A was compared among all six subclusters. Interestingly, subcluster 3 expressed intermediate FCGR3A, subcluster 6 expressed the highest FCGR3A, and little FCGR3A was detected in the remaining four subclusters (Figure 5I). In the dynamic expression profile of sd easily find that APOBEC3A became highly expressed at the final stage which is labelled with FCGR3A positive (Figure 5J).

We also generated a correlation heatmap to illustrate the relationships among APOBECs and different immune cell infiltration (absolute abundance, CIBERSORT outputs of TCGA) in bulk BLCA samples (Figure 5K). Among all cases, we observed that the pair of APOBEC3A-M1 exhibited the highest correlation (r = 0.334, p = 5.09e-12). The cumulative proportion curves (Figure 5L) and violin plot (Figure 5M) jointly demonstrated that a significantly higher abundance of M1 infiltration was observed in BLCA samples with higher APOBEC3A expression. These evidences from scRNA-seq and bulk RNA-seq data demonstrated that APOBEC3A may play a role in the differentiation of monocytes in BLCA patients.

### AMES correlates with immune infiltration and potential ICB response in BLCA

**Figure 6:**
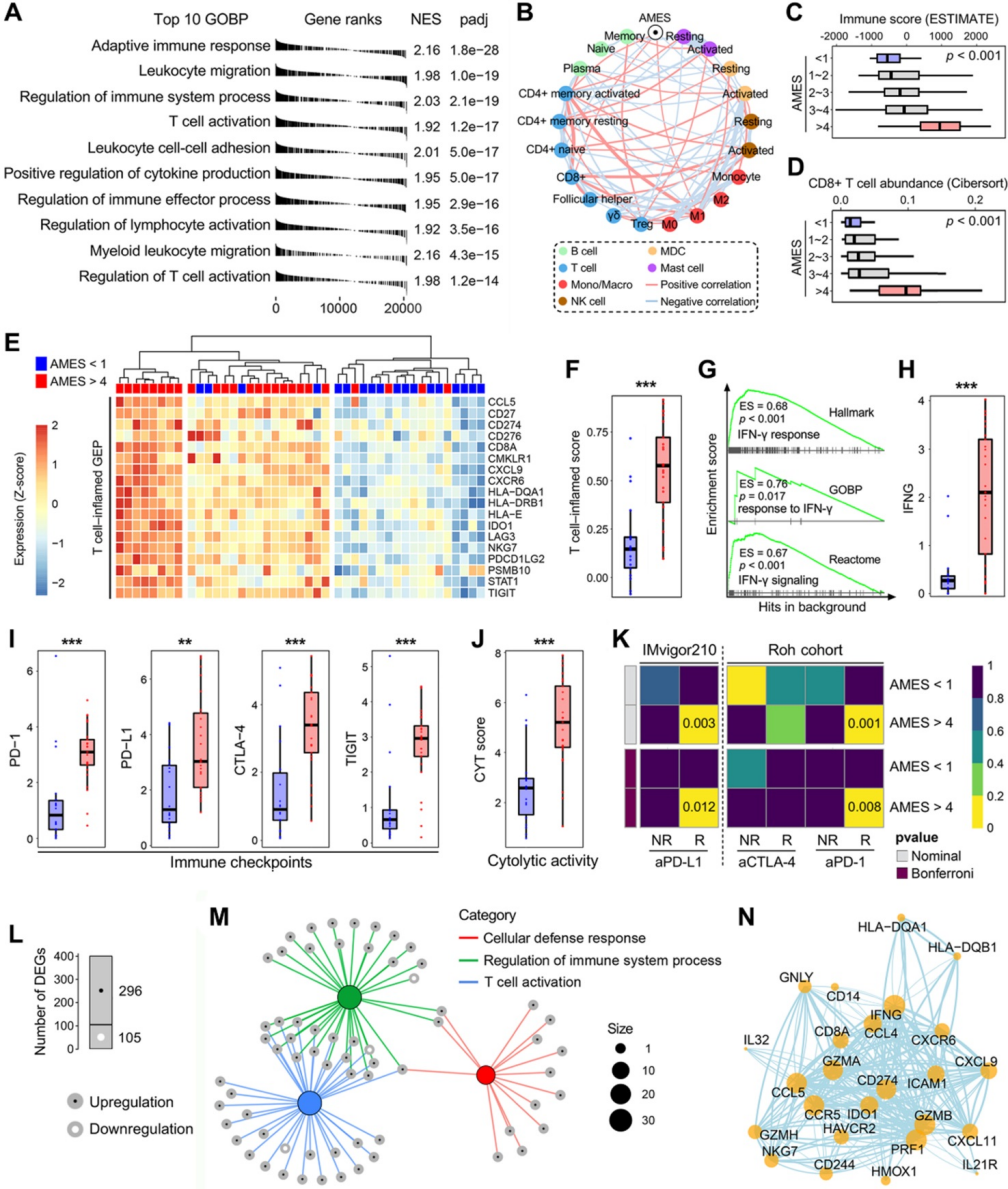
** Higher AMES confers higher immune infiltration and potential ICB response in BLCA. (A)** A fgsea algorithm was performed with all GOBP gene sets in different AMES groups, and the most significantly altered pathway was annotated as “adaptive immune response” in the AMES-high group (NES = 2.16, p = 1.8e-28). **(B)** A correlation network depicts the relationships among AMES and various immune cell types in bulk samples. AMES held significantly positive correlations with activated CD4+ memory T cell, CD8+ T cell and M1 abundance, while negatively correlated with CD4+ naïve and activated MDC populations. **(C)** As AMES level increased, the immune infiltration score was stepwisely and significantly elevated, **(D)** as well as the CD8+ T cell abundance. **(E)** A T cell-inflamed GEP (18 genes) which is correlated with ICB response was introduced to evaluate the predictive potential of AMES for cancer immunotherapy. As shown in the heatmap, the T cell-inflamed GEP holds extensively high expressions in samples with the highest AMES compared to those with the lowest AMES, **(F)** and ssGSEA algorithm validated that the T cell-inflamed score was significantly elevated in the AMES > 4 group. **(G)** GSEA was performed with three relevant but independent gene sets, and IFN-γ response was significantly enhanced in the AMES > 4 group. **(H)** IFNG mRNA expression (log2-normalized) is significantly elevated in the AMES > 4 group. **(I)** Representative immune checkpoints including PD-1, PD-L1, CTLA-4, and TIGIT were extensively elevated in the AMES > 4 group, **(J)** as well as the CYT score. **(K)** SubMap analysis revealed that the AMES > 4 group exhibited a high likelihood of response to ICB when compared to two immunotherapy cohorts (IMvigor210 and Roh's cohort). **(L)** With a threshold of FDR q < 0.01, a total of 401 DEGs (296 upregulated and 105 downregulated genes) were identified in the AMES > 4 group. **(M)** Most DEGs were enriched in immune-related pathways. **(N)** A PPI network reveals interactions among representative DEGs based on the STRING database.

To investigate the biological properties correlated with AMES in BLCA, we performed a fgsea algorithm with all GOBP gene sets in different AMES groups (Figure 6A). Samples with higher AMES exhibited significantly higher activity of various immune responses, and the most significantly altered pathway was annotated as “adaptive immune response” (NES = 2.16, p = 1.8e-28; Figure 6A). A correlation network was generated to depict the relationships among AMES and various immune cell types in bulk samples, in which pink lines represent positive correlation and lightblue lines represent negative correlation. In particular, we found that AMES held significantly positive correlations with activated CD4+ memory T cell, CD8+ T cell, and M1 abundance, while negatively correlated with CD4+ naïve and activated MDC populations (Figure 6B). In addition, as AMES level increased, the immune infiltration score was stepwisely and significantly elevated (Figure 6C), as well as the CD8+ T cell abundance (Figure 6D). Then we focused on AMES < 1 (lowest) and > 4 (highest) groups to investigate the intrinsic differences of immune features. A T cell-inflamed GEP (18 genes) which is correlated with ICB response was introduced to evaluate the predictive potential of AMES for cancer immunotherapy. As shown in the heatmap (Figure 6E), the T cell-inflamed GEP holds extensively high expressions in samples with the highest AMES compared to those with the lowest AMES, and ssGSEA algorithm validated that the T cell-inflamed score was significantly elevated in the AMES > 4 group (Figure 6F). Considering IFN-γ is a cytokine that plays a critical role in immune regulation and anti-cancer immunity, we performed GSEA with three relevant but independent gene sets and found that IFN-γ response was significantly enhanced in the AMES > 4 group (Figure 6G). As expected, IFNG mRNA expression (log2-normalized) is also significantly elevated in the AMES > 4 group (Figure 6H).

Considering cancer immunotherapy with ICB is based on the inhibition of critical immune checkpoints, we evaluated some representative molecules and found that PD-1, PD-L1, CTLA-4, and TIGIT were extensively elevated in the AMES > 4 group (Figure 6I), as well as the CYT score which is used to reflect the cytotoxic effects (Figure 6J). Furthermore, the SubMap analysis revealed that the AMES > 4 group exhibited a high likelihood of response to ICB, including anti-PD-L1, PD-1, and CTLA-4 in two immunotherapy cohorts (IMvigor210 and Roh's cohort; Figure 6K).

With a threshold of FDR q < 0.01, a total of 401 DEGs (296 upregulated and 105 downregulated genes; see details in supplementary table 2) were identified in the AMES > 4 group (Figure 6L). Most DEGs were enriched in immune-related pathways, and the three most significant biological processes were annotated as “cellular defense response”, “regulation of immune system process”, and “T cell activation” (Figure 6M). Based on the STRING database, a PPI network was generated to reveal interactions among representative DEGs (Figure 6N). Overall, these findings demonstrated that AMES correlates with immune activation and indicates potential ICB benefits in BLCA.

### Higher AMES predicts better prognosis in BLCA

**Figure 7:**
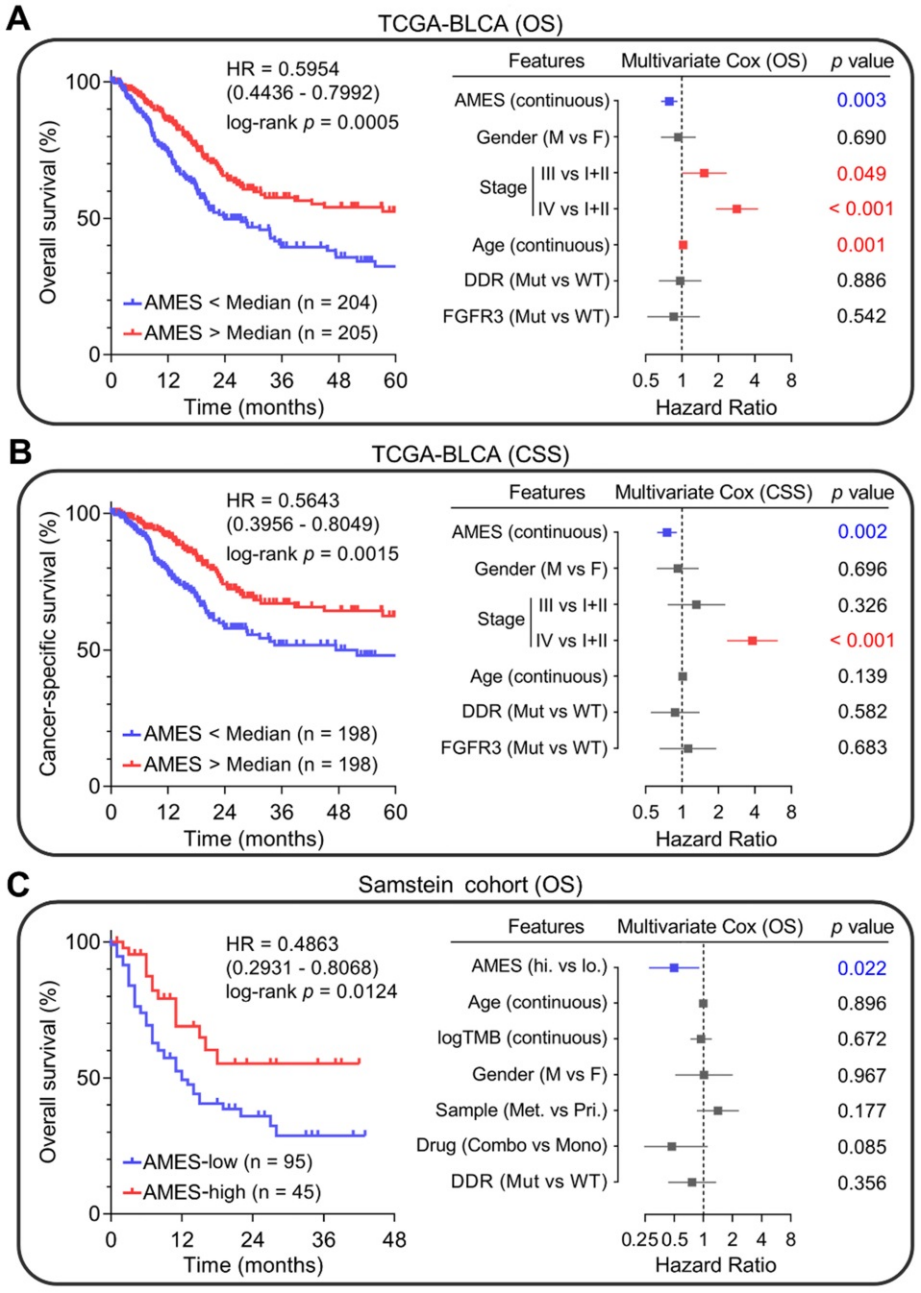
** Higher AMES predicts better prognosis in BLCA. (A)** Kaplan-Meier analysis showed that patients with higher AMES exhibited better overall survival (OS) in the TCGA-BLCA cohort (HR = 0.5954, 95% CI = 0.4436-0.7992, p = 0.0005; left panel). Multivariate Cox regression analysis indicated that among various clinicopathological features, AMES acts as the only independent protective factor (p = 0.003). Meanwhile, advanced pathological stage and elder act as independent risk factors for OS (right panel). **(B)** For cancer-specific survival (CSS), AMES still retains a positive correlation with a better prognosis (HR = 0.5643, 95% CI = 0.3956 - 0.8049, p = 0.0015; left panel). In the multivariate Cox regression analysis, AMES still serves as a protective factor (p = 0.002; right panel). **(C)** In Samstein's cohort of 140 advanced BLCA patients who received ICB therapy, Kaplan-Meier analysis showed that higher AMES correlated with better OS (HR = 0.4863, 95% CI = 0.2931-0.8068, p = 0.0124; left panel), and multivariate Cox regression analysis further demonstrated that higher AMES acts as the only protective factor for OS (p = 0.022), even outperforms some conventional predictors such as TMB, DDR status, and combination ICB therapy (right panel).

Next, we evaluated the prognostic value of AMES in BLCA. Kaplan-Meier analysis showed that patients with higher AMES exhibited more favorable overall survival (OS) in the TCGA-BLCA cohort (HR = 0.5954, 95% CI = 0.4436 - 0.7992, p = 0.0005; left panel of Figure 7A). Furthermore, multivariate Cox regression analysis indicated that among various clinicopathological features, AMES acts as the only independent protective factor (p = 0.003). Meanwhile, advanced pathological stage and elder act as independent risk factors for OS (right panel of Figure 7A). For CSS, AMES still retains a positive correlation with a better prognosis (HR = 0.5643, 95% CI = 0.3956-0.8049, p = 0.0015; left panel of Figure 7B). In the multivariate Cox regression analysis, AMES still serves as a protective factor (p = 0.002), and advanced pathological stage as the only risk factor for CSS (right panel of Figure 7B).

Subsequently, we evaluated whether AMES could serve as a promising biomarker for ICB. In Samstein's cohort of 140 advanced BLCA patients who received ICB therapy 26, Kaplan-Meier analysis showed that higher AMES correlated with better OS (HR = 0.4863, 95% CI = 0.2931-0.8068, p = 0.0124; left panel of Figure 7C), and multivariate Cox regression analysis further demonstrated that higher AMES acts as the only protective factor for OS (p = 0.022), better than some conventional predictors such as TMB, DDR status, and combination ICB therapy (right panel of Figure 7C). These findings suggested that AMES challenges some conventional biomarkers for survival prediction of BLCA patients, especially for those who received ICB treatment.

### A prognostic APOBEC mutagenesis-related model was established for BLCA patients

**Figure 8:**
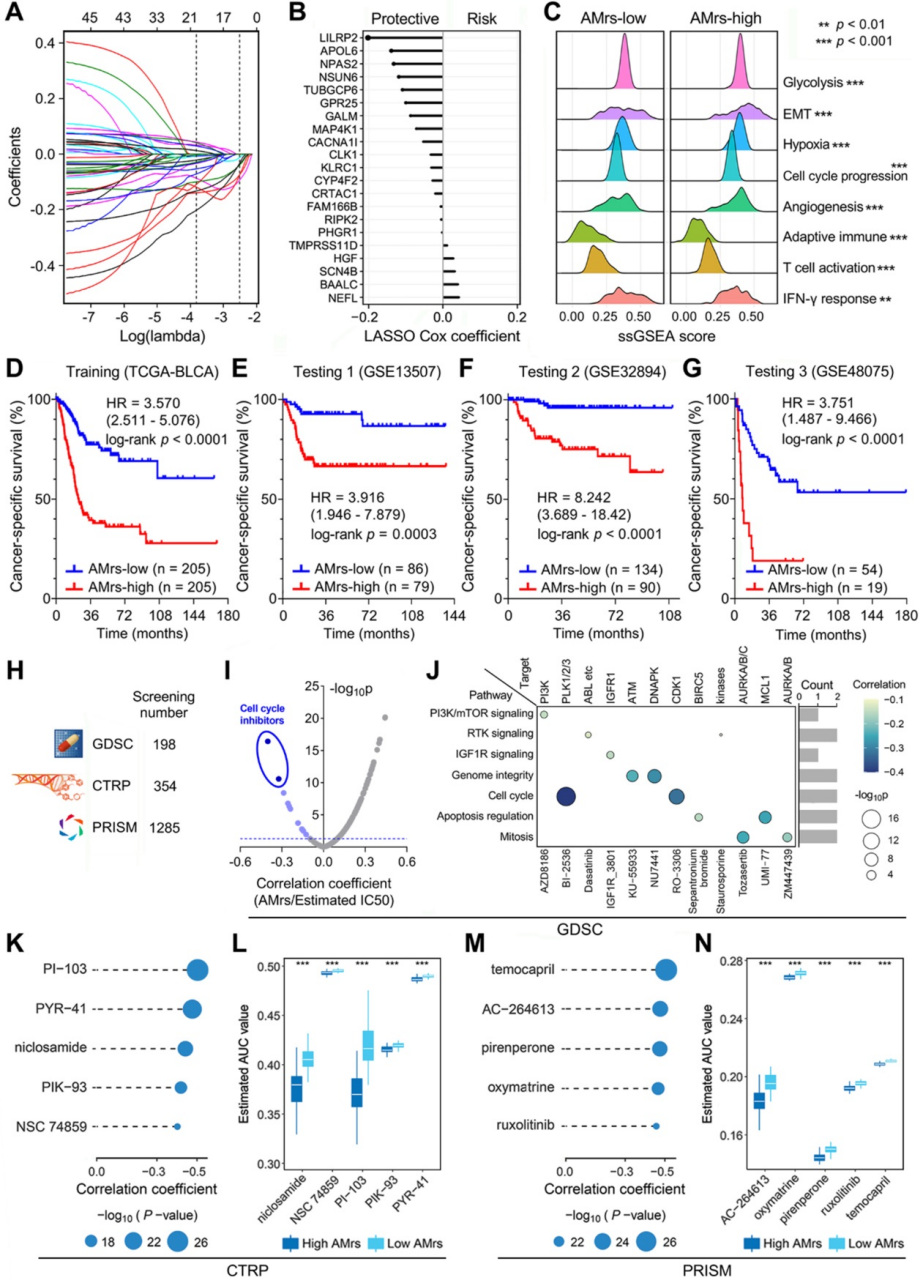
** A prognostic APOBEC mutagenesis-related model was established and validated, and potentially applicable drugs were screened for the high-risk subset. (A & B)** After LASSO regularization (10-fold cross-validation, optimal λ = 0.022), 21 genes retained their Cox coefficients, and a prognostic APOBEC mutagenesis-related risk score (AMrs) was calculated for each BLCA patient. **(C)** The ridgeline plots showed that significant differences in the performances of various cancer hallmarks were observed between AMrs-low and AMrs-high samples. **(D)** In the training set (TCGA-BLCA), patients with higher AMrs exhibited worse CSS (HR = 3.570, 95% CI = 2.511-5.076, p < 0.0001). **(E-G)** The prognostic value of AMrs was validated for CSS in three independent BLCA cohorts (GSE13507: HR = 3.916, 95% CI = 1.946-7.879, p = 0.0003; GSE32894: HR = 8.242, 95% CI = 3.689-18.42, p < 0.0001; GSE48075: HR = 3.751, 95% CI = 1.487-9.466, p < 0.0001). **(H)** A total of 1,837 compounds from three drug response databases (GDSC, CTRP, and PRISM) were screened to identify potential therapeutic targets and compounds for patients with high AMrs. **(I)** For the GDSC database, Spearman correlation analysis was performed on AMrs and estimated IC50 values. With a filtering threshold of negative r value and p value less than 0.05, 12 candidate compounds were identified, and two compounds with most negative correlation coefficients were annotated as cell cycle inhibitors, namely BI-2536 and RO-3306. **(J)** The signaling pathways and therapeutic targets of the 12 candidate compounds from GDSC. **(K & M)** AUC values of compounds from CTRP and PRISM were estimated for each TCGA sample, and Spearman correlation analysis was performed on AMrs and estimated AUC values. For both CTRP and PRISM, five compounds with most negative correlation coefficients were displayed in dot-line plots (CTRP: PI-103, PYR-41, niclosamide, PIK-93, NSC 74859; PRISM: temocapril, AC-264613, pirenperone, oxymatrine, ruxolitinib), **(L & N)** and all estimated AUC values of these compounds were significantly lower in the AMrs-high group. *** p < 0.001.

Considering AMES could predict survival well, we attempted to construct an AMES-based gene signature for individual risk assessment of CSS. Firstly, the 401 aforementioned DEGs were submitted for univariate Cox regression analysis, and 45 candidates were filtered with a threshold p value less than 0.01. After LASSO regularization (10-fold cross-validation, optimal λ = 0.022; Figure S5 & Figure 8A), 21 genes retained their Cox coefficients (Figure 8B; Supplementary Table 3), and a prognostic APOBEC mutagenesis-related risk score (AMrs) was calculated for each BLCA patient as described in the methods section. The ridgeline plots showed that significant differences in the performances of various cancer hallmarks were observed between AMrs-low and AMrs-high samples (Figure 8C). In the training set (TCGA-BLCA), patients with higher AMrs exhibited worse CSS (HR = 3.570, 95% CI = 2.511-5.076, p < 0.0001; Figure 8D). The prognostic value of AMrs was validated for CSS in three independent BLCA cohorts (GSE13507: HR = 3.916, 95% CI = 1.946-7.879, p = 0.0003; GSE32894: HR = 8.242, 95% CI = 3.689-18.42, p < 0.0001; GSE48075: HR = 3.751, 95% CI = 1.487-9.466, p < 0.0001) (Figure 8E-G). In addition, in a cohort of 460 NMIBC patients, AMrs still retained its prognostic capacity for progression-free survival, with a HR = 5.509 (95% CI = 2.624-11.56, p < 0.0001; Figure S6). Details of AMrs and follow-up information of TCGA and four validation cohorts could be found in Supplementary Table 4. These results demonstrated that AMrs could function as an ideal prognostic tool for BLCA patients.

### Drug sensitivity analysis for BLCA patients with different AMrs

Potential druggable targets and corresponding compounds that are highly correlated with AMrs may have useful therapeutic implications for high-risk BLCA patients. To identify potential therapeutic targets and compounds for patients with high AMrs, we screened a total of 1,837 compounds from three drug response databases (GDSC, CTRP, and PRISM; Figure 8H). Firstly, IC50 values of 198 compounds from GDSC were estimated for each TCGA sample, and Spearman correlation analysis was performed on AMrs and estimated IC50 values. With a filtering threshold of negative r value and p value less than 0.05, 12 candidate compounds were identified, and two compounds with the most negative correlation coefficients were annotated as cell cycle inhibitors, namely BI-2536 and RO-3306 (Figure 8I). In detail, the signaling pathways and therapeutic targets of the 12 candidate compounds were summarized in Figure 8J. AUC values of compounds from CTRP and PRISM were estimated for each TCGA sample, and Spearman correlation analysis was performed on AMrs and estimated AUC values. For both CTRP and PRISM, five compounds with the most negative correlation coefficients were displayed in dot-line plots (CTRP: PI-103, PYR-41, niclosamide, PIK-93, NSC 74859; PRISM: temocapril, AC-264613, pirenperone, oxymatrine, ruxolitinib) (Figure 8K & M), and their estimated AUC values were compared in different AMrs groups (Figure 8L & N). Overall, all these identified compounds have a significantly negative correlation with AMrs and lower estimated AUC values in the high-AMrs group.

## Discussion

The APOBEC family acts as a double-edged sword towards humans due to their intrinsic ability of cytosine deamination in genome. On the one hand, APOBECs act as antiviral factors via mutation of viral genomes thereby restricting viruses and reducing infectivity; on the other hand, they also act as DNA mutators which play an important role in tumorigenesis and cancer evolution 52. However, the role of APOBECs in cancers remains ambiguous. For example, it is reported that APOBEC mutagenesis could drive tumor evolution in metastatic thoracic tumor and HPV-driven tumor but inhibit breast cancer growth through immune activation 12, 53, 54. When it comes to APOBEC3B, it is the most widely investigated member of APOBEC family and causes a variety of mutagenic outcomes 55, but its biological impact on cancers still remains unclear, even contrary. For example, Xia and colleagues reported that APOBEC3B upregulation predicts immune inactivation and worse survival in gastric cancer 56, while Serebrenik and colleagues reported that APOBEC3B is overexpressed in a subset of clear cell ovarian cancer and correlates with improved clinical outcomes 57. As regards to cancer therapy, APOBEC3B promotes tamoxifen resistance in ER-positive breast cancer but predicts favorable response of ICB in NSCLC 14, 15. Taken together, to gain a more comprehensive knowledge of APOBEC family in pan-cancer and to reveal inherent characteristics in some specific cancer types is challenging but warranted.

The TCW mutation is predominantly caused by APOBEC enzymatic activities 9. In this study, we calculated the APOBEC mutagenesis enrichment score (AMES) to quantify this mutational pattern by adjusting TCW mutations in the mutation background. We believe AMES is more reasonable to evaluate the APOBEC mutagenesis than direct counting of TCW mutations because some certain cancer type with high total mutation burden, such as SKCM, definitely has more TCW mutations, but the relative abundance of APOBEC-mediated mutagenesis is not really high (compared to other cancer types). McGrail and colleagues claimed that TMB fails to predict ICB response across all cancer types and suggested more attention should be focused on cancer type-specific assessment of TMB 58. A cancer type-specific TMB threshold will certainly improve the prediction of ICB response, but we hold an opinion that the distribution of cancer type-specific or ICB sensitivity-specific mutational pattern is a more ideal and individualized predictor for ICB response. TMB calculation contains a wide range of non-silent mutation variants, but these mutations contribute differently to immune activation and ICB response. Using TMB as a predictor for ICB still remains in the concept of “quantity of mutation”, which is undoubtedly a rough index and far from personalized treatment. By contrast, we think the abundance of ICB sensitivity-specific mutational patterns is a step towards “quality of mutation”, which could individually reflect the potential response of ICB therapy.

Plenty of evidence was presented to support this viewpoint in our study. Firstly, the APOBEC mutagenesis was identified as a dominant mutational pattern in BLCA, and it is mostly correlated with TMB among all solid cancer types. Using the quantitative method mentioned above, we calculated the AMES for each BLCA sample, and we observed that higher AMES correlates with higher immune infiltration, CD8+ T cell activation, and IFN-γ response. In addition, the expression levels of immune checkpoints (PD-1, PD-L1, CTLA-4, and TIGIT) and potential cytolytic activity were significantly elevated in the AMES-high group. Further, AMES-high samples exhibited a high similarity of the transcriptional profile with samples that were responsive to ICB, indicating the potential response of AMES-high BLCA samples to ICB therapy. In addition to bioinformatic evidence, AMES serves as a promising biomarker for prognosis in the clinical setting. In the TCGA-BLCA cohort, higher AMES predicts better overall survival and cancer-specific survival and is the only protective factor for survival in the multivariate Cox regression analysis. Notably, in a cohort of advanced BLCA patients who received ICB therapy, AMES is the only significant parameter (protective factor) which correlates with overall survival, even outperforms some traditional features such as TMB and DDR status (Figure 7C). According to the clinical consensus, DDR status is a binary variable defined as mutation or wild-type, and its alteration is associated with favorable survival in advanced BLCA patients 51, 59. Intriguingly, in this study, AMES offers advantages over DDR in the prediction of ICB response, and a potential explanation is that APOBECs contributed a lot not only to the mutations of DDR genes (Figure 2I & J), but also other mutations which contribute substantially to cancer immunogenicity and trigger immune activation in BLCA. In short, we speculated that APOBEC mutagenesis could induce more neoantigens and enable BLCA to become more sensitive to ICB than DDR mutation alone. Of course, more clinical trials are needed to further confirm the predictive value of AMES in prognosis and ICB response.

Considering AMES could predict clinical outcomes well, we attempted to construct a prognostic APOBEC mutagenesis-related model using machine learning approaches, and this model functions well in different BLCA cohorts. In addition, potential druggable targets and corresponding compounds were screened for BLCA patients who are defined as high-risk with the established prognostic model, and the two most promising compounds, namely BI-2536 and RO-3306, were identified from the GDSC drug response database. Interestingly, both of them are cell cycle inhibitors.

Single-cell RNA-sequencing datasets were used to delineate the expression landscape and biological functions of APOBECs in BLCA. We observed that APOBEC3B is not only upregulated in malignant epithelial cells compared to normal, but also correlates with the malignant progression in BLCA. As regards to APOBEC3A, it is specifically expressed in monocytes, and may have a crucial role in the differentiation from classical monocytes to FCGR3A+ monocytes and to M1 macrophages. These findings demonstrated that APOBEC3A and 3B play a significant role within the tumor microenvironment of BLCA, especially in the malignant evolution and cell differentiation.

## Conclusions

Altogether, our systematic analysis revealed expression profiles of APOBECs and distribution characteristics of APOBEC mutagenesis in pan-cancer, and further reported that APOBEC mutagenesis could be a potential biomarker for survival prediction and immunotherapy response for BLCA patients. Nevertheless, more clinical and experimental studies are expected to validate these findings.

## Supplementary Material

Supplementary figures and table legends.

Supplementary table 1.

Supplementary table 2.

Supplementary table 3.

Supplementary table 4.

## Abbreviations

TCGA: The Cancer Genome Atlas
TCIA: The Cancer Immunome Atlas
GEO: Gene Expression Omnibus
GTEx: Genotype-Tissue Expression
CCLE: Cancer Cell Line Encyclopedia
GDSC: Genomics of Drug Sensitivity in Cancer
CTRP: Cancer Therapeutics Response Portal
PRISM: Profiling Relative Inhibition Simultaneously in Mixtures
ACC: Adrenocortical Cancer
BLCA: Bladder Cancer
BRCA: Breast Cancer
CESC: Cervical Cancer
CHOL: Bile Duct Cancer
COAD: Colon Cancer
ESCA: Esophageal Cancer
GBM: Glioblastoma
HNSC: Head and Neck Cancer
KICH: Kidney Chromophobe
KIRC: Kidney Clear Cell Carcinoma
KIRP: Kidney Papillary Cell Carcinoma
LGG: Lower Grade Glioma
LIHC: Liver Cancer
LUAD: Lung Adenocarcinoma
LUSC: Lung Squamous Cell Carcinoma
MESO: Mesothelioma
OV: Ovarian Cancer
PAAD: Pancreatic Cancer
PRAD: Prostate Cancer
READ: Rectal Cancer
SARC: Sarcoma
SKCM: Melanoma
STAD: Stomach Cancer
TGCT: Testicular Cancer
THCA: Thyroid Cancer
THYM: Thymoma
UCEC: Endometrioid Cancer
NSCLC: Non-Small Cell Lung Cancer
APOBEC: Apolipoprotein B mRNA editing enzyme, catalytic polypeptide-like
ANT: Adjacent normal tissue
CSS: Cancer-specific survival
OS: Overall survival
WES: Whole-exome sequencing
NGS: Next-generation sequencing
ICB: Immune checkpoint blockade
GSEA: Gene set enrichment analysis
DDR: DNA damage response
RF: Random forest
LASSO: Least absolute shrinkage and selection operator
MSigDB: Molecular Signatures Database
TMB: Tumor mutation burden
TNB: Tumor neoantigen burden
ITH: Intra-tumor heterogeneity
scRNA-seq: Single-cell RNA-sequencing
DEGs: Differentially expressed genes
FDR: False discovery rate
CYT: Cytolytic activity
AMES: APOBEC mutagenesis enrichment score
AMrs: APOBEC mutagenesis-related risk score
