# Supplementary material for: APOBEC-mediated mutagenesis is a favorable predictor of prognosis and immunotherapy for bladder cancer patients: evidence from pan-cancer analysis and multiple databases
Source: Theranostics. 2022 May 16;12(9):4181–99. doi: 10.7150/thno.73235 (PMC9169361; doi:10.7150/thno.73235)
Supplement: Supplementary file 1 — Supplementary figures and table legends. [file thnov12p4181s1.pdf]

Supplementary figures and legends:

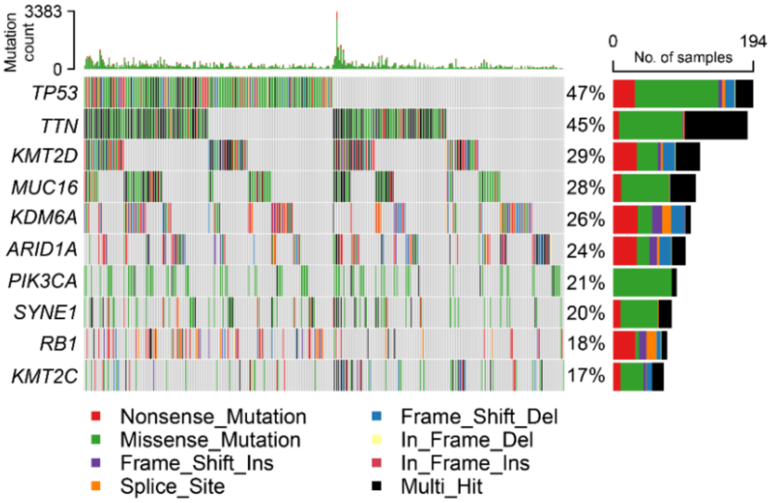

Figure S1: An oncoplot shows the 10 most frequent non-silent mutations (TP53, TTN, KMT2D, MUC16, KDM6A, ARID1A, PIK3CA, SYNE1, RB1, and KMT2C) in BLCA.

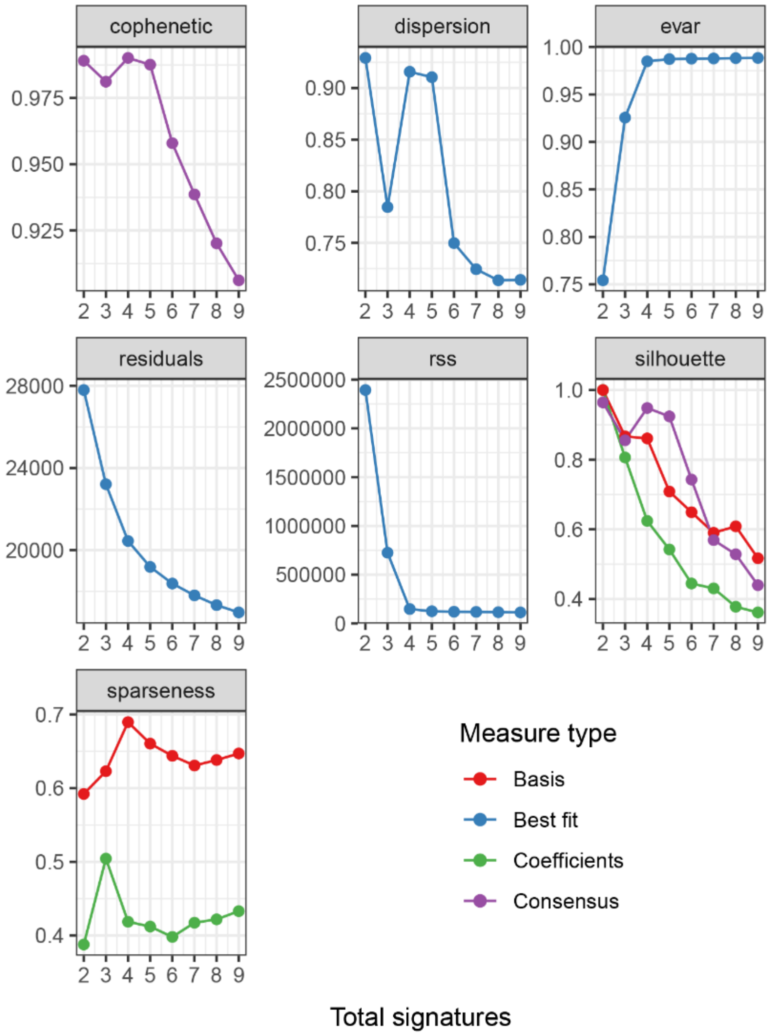

Figure S2: In the NMF algorithm, the optimal factorization of k value (k = 5) was selected when the magnitude of the cophenetic correlation coefficient begins to fall.

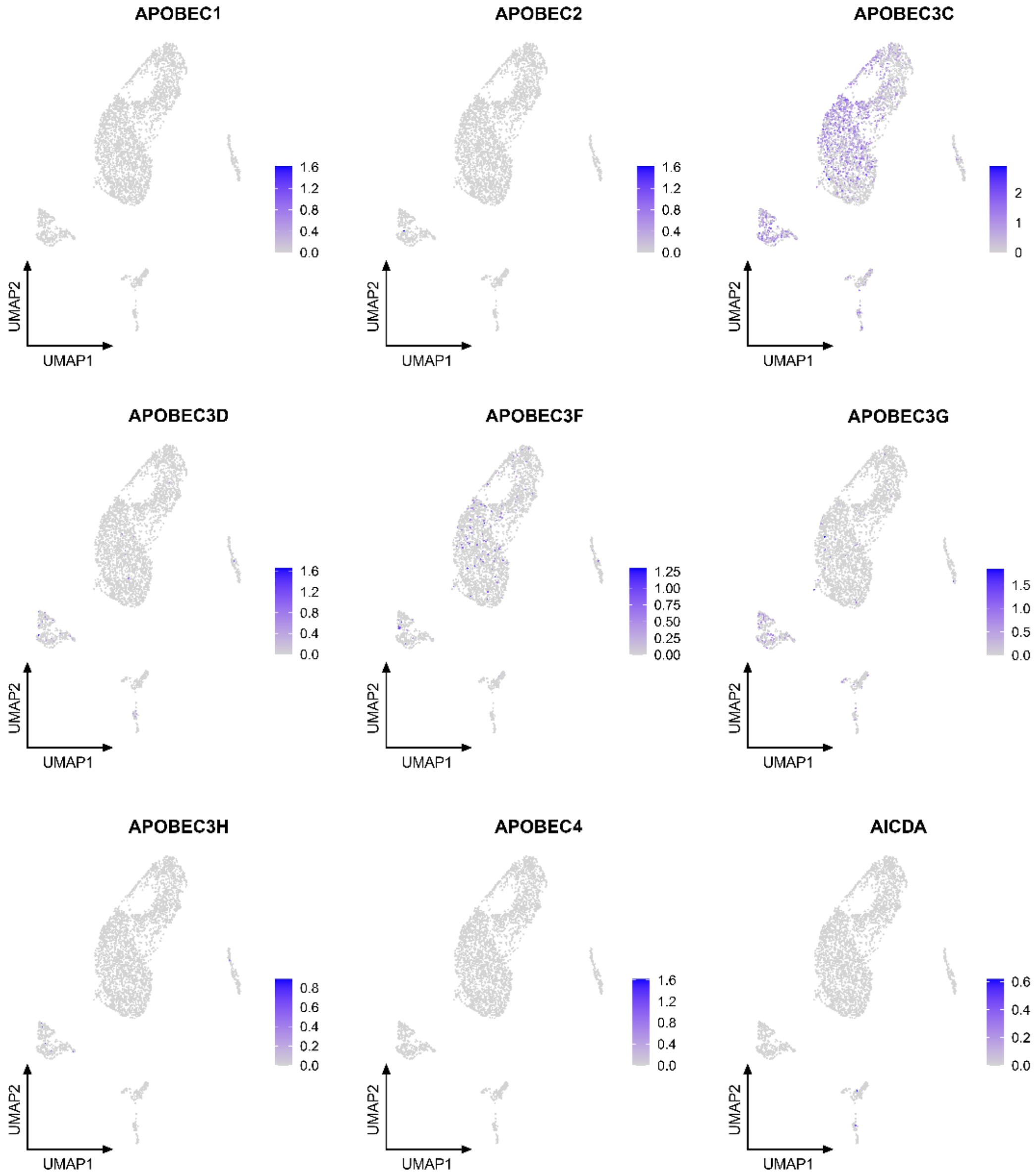

Figure S3: Expression landscape of APOBECs in GSE130001.

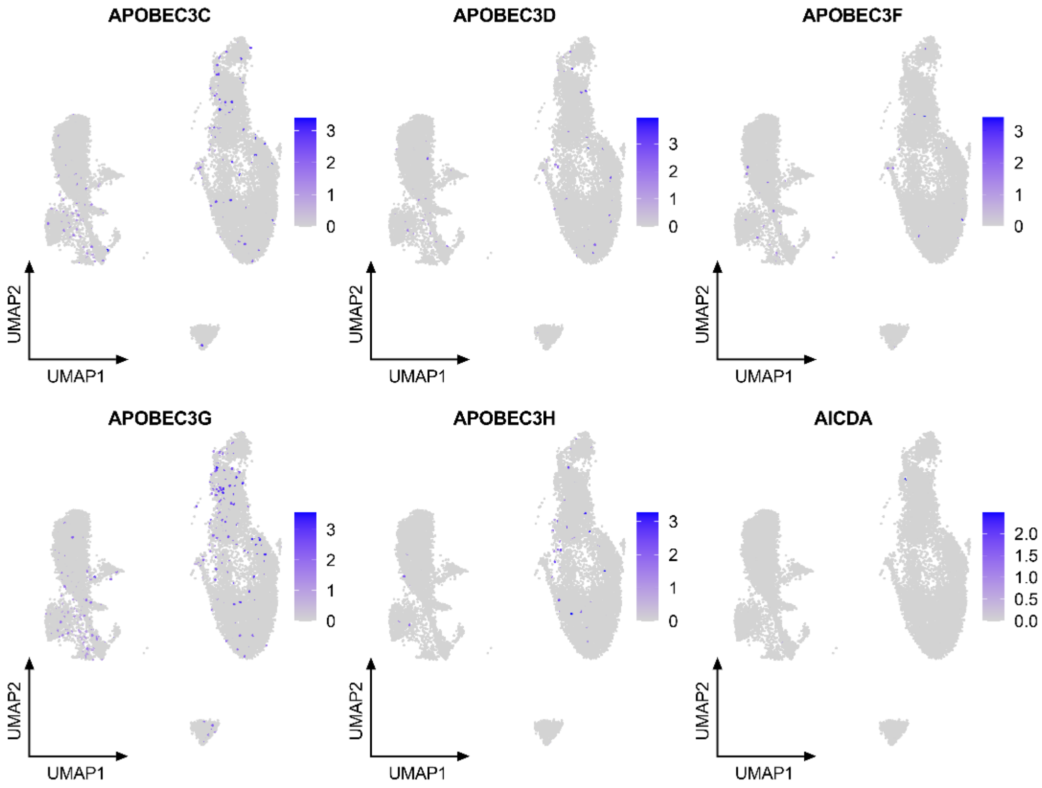

Figure S4: Expression landscape of APOBECs in GSE145281.

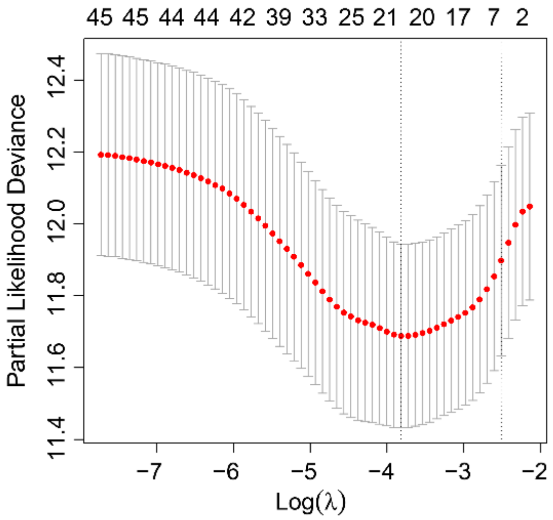

Figure S5: The optimal penalization coefficient (λ = 0.022) in the LASSO model was identified by 10-fold cross-validation.

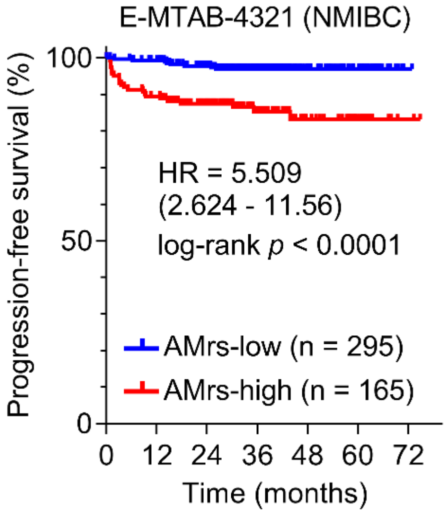

Figure S6: In a cohort of 460 non-muscle invasive bladder cancer (NMIBC) patients, AMrs still retained a powerful prognostic capacity for progression-free survival, with a HR = 5.509 (95% CI = 2.624 – 11.56, p < 0.0001).

Legends of supplementary tables:

sTable1: APOBEC mutagenesis enrichment score (AMES) of pan-cancer samples in TCGA.

sTable2: A list of 401 differentially expressed genes (DEGs).

sTable3: LASSO Cox coefficients of 21 prognostic genes.

sTable4: AMrs and follow-up information of training cohort (TCGA) and four validation cohorts (GSE13507, GSE32894, GSE48075, E-MTAB-4321).
